# The Hidden Cost of Modern Medical Interventions: How Medical Advances Have Shaped the Prevalence of Human Fungal Disease

**DOI:** 10.3390/pathogens8020045

**Published:** 2019-04-04

**Authors:** Callum Clark, Rebecca A. Drummond

**Affiliations:** Institute of Immunology & Immunotherapy, Institute of Microbiology & Infection, University of Birmingham, Birmingham B15 2TT, UK; callum.clark@nottingham.ac.uk

**Keywords:** fungi, antibiotics, corticosteroids, ibrutinib, Candida, Aspergillus, macrophage

## Abstract

Life expectancy in the West is the highest it has ever been, due to the introduction of better hygiene practices and sophisticated medical interventions for cancer, autoimmunity and infectious disease. With these modern advances, a rise in the prevalence of opportunistic infections has also been observed. These include several fungal infections, which present a particular clinical challenge due to the lack of fungal vaccines, limited diagnostics and increasing antifungal drug resistance. This mini-review outlines how modern-day clinical practices have shaped the recent increase in fungal diseases observed in the last few decades. We discuss new research that has implicated the use of immune-modulating drugs in the enhanced susceptibility of vulnerable patients to life-threatening fungal infections.

## 1. Introduction

Fuelled by a wealth of new technological and pharmaceutical advances, and an improved focus on the importance of hygiene and vaccination, human life expectancy has increased by over 50% over the last century [1]. Moreover, the advancement of recombinant DNA technology and the development of new biologics to generate safer vaccines and specific monoclonal antibodies has also become increasingly possible, leading to the development of more targeted therapies, such as small-molecule inhibitors (e.g. receptor tyrosine kinase inhibitors) and monoclonal antibody anti-cancer therapeutics. Alongside the introduction of these advances, the prevalence of life-threatening fungal infections has increased, which has largely coincided with significant progress in the treatment of other comorbidities. For example, the development of immunomodulatory agents for the control of cancer and treatment of autoimmunity has led to a significant increase in the number of immunosuppressed patients who are susceptible to opportunistic infections. The majority of fungal infections are opportunistic, since healthy individuals are able to mount successful immune responses against these microbes and rarely develop clinical infection. Deficiencies in the immune system (e.g., neutropenia), caused by chemotherapy conditioning regimens or immunomodulatory agents, render patients vulnerable to fungi, which they easily become exposed to, due to the large concentration of fungal spores in the environment and within the human commensal flora.

**Figure 1:**
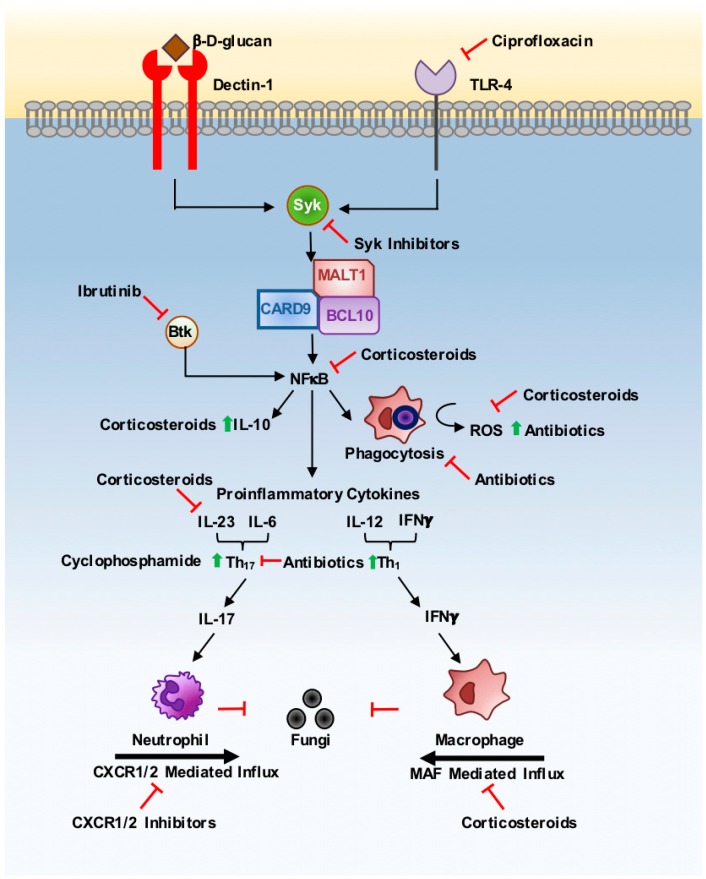
Iatrogenic interference with the initiation of antifungal immunity. Pathogen-associated molecular patterns (PAMPs) in the fungal cell wall (e.g., β-glucan) are detected by pattern recognition receptors (PRRs) on the plasma membranes of host phagocytes (e.g., Dectin-1). This initiates Syk-dependent assembly of the CBM (CARD9-BCL10-MALT1) signalosome, leading to NF-κB activation, mitogen-activated protein kinase (MAPK) signalling and a calcium flux, resulting in antifungal cellular responses. Treatments for comorbidities, such as anti-cancer therapies plus antibiotics, can interfere with this fungal detection pathway, as highlighted by red inhibitory arrows. Syk, spleen tyrosine kinase; CARD9, caspase recruitment domain-containing protein 9; MALT1, mucosa associated lymphoid tissue translocation protein 1; BCL10, B-cell lymphoma 10; IL, interleukin; Th, T helper; MAF, macrophage-activating factor; ROS, reactive oxygen species; NFκB, nuclear factor kappa B.

Invasive fungal infections kill over 1.5 million individuals annually [2]. Fungal infections may also be self-limiting mucosal/skin infections, such as oropharyngeal candidiasis, which, whilst non-lethal, can significantly reduce quality of life and impact the lives of >1 billion people [3]. Fungi are recognised by myeloid cells, such as neutrophils, macrophages and dendritic cells (DCs), via host pattern recognition receptors (PRRs), including the C-type lectin receptors (CLRs) and toll-like receptors (TLRs), which bind to pathogen-associated molecular patterns (PAMPs) embedded in the fungal cell wall [4]. The most critical antifungal immune molecule identified to date is CARD9, a signalling adaptor protein found downstream of several CLRs, including Dectin-1, which recognises the fungal cell wall carbohydrate β-glucan. Binding of CARD9-coupled CLRs initiates a signalling cascade via Syk kinase and the CBM (CARD9-BCL10-MALT1) signalosome to activate NF-κB, mitogen-activated protein kinase (MAPK) signalling and a calcium flux, resulting in pro-inflammatory cytokine release, phagocytosis and the killing of fungal cells (Figure 1). Dectin-1-CARD9 signalling is also thought to influence antigen presentation by DCs to shape adaptive immune responses, of which T_h_1 and T_h_17 responses are considered the most protective. Human mutations that result in disruption of these pathways cause enhanced susceptibility to fungal infections, underscoring their importance in antifungal immunity. For example, human CARD9 deficiency is associated with the spontaneous development of life-threatening *Candida* infections [5], while genetic mutations in Dectin-1 predispose individuals to chronic skin and nail fungal infections [6]. In this mini-review, we discuss how commonly prescribed drugs, such as steroids, antibiotics and biologic drugs, as well as emerging small-molecule inhibitors for anti-cancer therapies, affect these critical antifungal pathways that protect us against infection, and how their use has shaped the prevalence of human fungal diseases in modern times.

## 2. Corticosteroids

A major risk factor for invasive fungal infections are solid organ and stem cell transplants due to the immune-suppressing drug regimens used to prevent transplant rejection, which include the use of corticosteroids such as dexamethasone [7]. The most common fungal infections observed in transplant recipients are invasive aspergillosis and invasive candidiasis [7]. Invasive aspergillosis is most often caused by *Aspergillus fumigatus*, which invades the lung and central nervous system (CNS) following inhalation of spores, while invasive candidiasis is predominantly caused by *Candida albicans*, and mostly involves the kidney, liver and spleen, following dissemination of yeast via the bloodstream [8].

Corticosteroids activate the glucocorticoid receptor, which is directly antagonistic to NF-κB, a central transcription factor for pro-inflammatory cytokine production that is activated by Syk-CARD9 signalling during fungal infection [9]. Corticosteroid-induced disruption of NF-κB signalling can therefore significantly disrupt immune responses to fungal pathogens (Figure 1). Indeed, human mutations in *IKK*, the NF-κB activator, result in severe susceptibility to chronic mucocutaneous candidiasis, which is associated with an impairment in the production of cytokines that protect the mucosal barrier against fungal infection, including IL-17A, IL-22 and IFNγ [10]. Until recently, it was regarded that abrogation of NF-κB signalling was the predominant mode of corticosteroid-induced immune suppression. However, recent studies suggest that corticosteroids may have wider effects on different types of immune cells. For example, corticosteroids induce a state of hyporesponsiveness in DCs, impairing their ability to drive and shape T-cell immunity by limiting their capacity to secrete polarising cytokines IL-12 and IL-23, and by increasing their IL-10 secretion [11,12]. In other studies, steroids have been shown to cause CD4^+^ T-cell apoptosis in the gastrointestinal (GI) tract, which contributed towards the resulting immune dysregulation within the tissue [13]. CD4^+^ T-cells are particularly critical for protection against mucosal candidiasis and infections with the fungus *Cryptococcus neoformans*, as both of these fungal infections are prevalent in patients with dysregulated T-cell responses (e.g., chronic HIV infection). Indeed, these infections are also common in patients treated with corticosteroids [14]. The exact consequences of steroid-induced T-cell dysregulation for antifungal immunity have yet to be fully explored, but understanding how these drugs affect T-cell responses to pathogenic fungi will be important for the future development of therapies in patients receiving corticosteroid therapy, who are particularly at risk for dangerous fungal infections.

Corticosteroid treatment also has a profound effect on myeloid cells, including monocytes and polymorphonuclear neutrophils (PMN). Specifically, corticosteroids impair the generation of reactive oxygen species (ROS) in PMN in response to opsonised *Escherichia coli*, via decreased levels of NADPH activation. Other pathways were also thought to be affected in this study, but the exact mechanisms have yet to be fully defined, so this will be a key area for future investigation [15]. The generation of ROS forms a key part of several fungi-killing pathways, since patients with defective ROS generation (chronic granulomatous disease, CGD) have a decreased ability of host phagocytes to kill *Aspergillus* and *Candida* species, leading to an increased risk of infections with these fungi in CGD patients [10]. Corticosteroids have also been shown to block macrophage-activating factor (MAF) release and macrophage chemotaxis, and can inhibit neutrophil apoptosis pathways, leading to defects in their maturation and function [16]. Despite the key role for neutrophils in the control and elimination of *Candida* spp. and *Aspergillus* spp., there have been few studies performed to determine the specific effects of corticosteroid treatment on the antifungal functions of these cells. Yet, this will help our understanding of how to reduce the risk of invasive fungal infections currently associated with corticosteroid use.

## 3. Antibiotics

**Figure 2:**
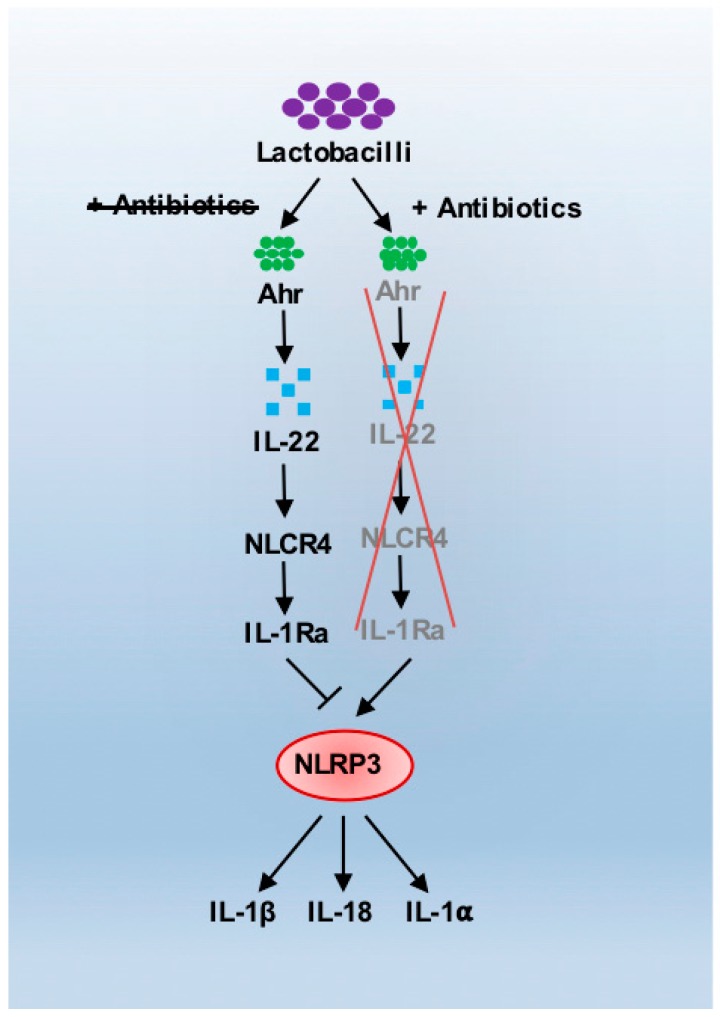
Antibiotics affect lactobacilli-mediated modulation of NLRP3 activation, predisposing to vulvovaginal candidiasis (VVC). Lactobacilli induce AhR (aryl hydrocarbon receptor)-dependent IL-22 (interleukin-22) transcription, promoting barrier function and regulation of mucosal immune responses through NLRC4-mediated production of the IL-1 receptor antagonist (IL1Ra), which inhibits activation of the NLRP3 inflammasome and production of IL-1β, IL-1α and IL-18, preventing neutrophil recruitment and *C. albicans* infection. Antibiotic treatment eradicates lactobacilli from the vaginal tract, leading to NLRP3 inflammasome activation and pro-inflammatory cytokine release, leading to dysbiosis and infection.

Broad-spectrum antibiotics are prescribed to multiple groups of patients at risk of bacterial infection, such as those in the intensive care unit (ICU), transplant patients and patients with acquired immunodeficiency (e.g., HIV). The increased usage of and dependence on antibiotics within our modern healthcare systems has led to a massive increase in bacterial resistance to these drugs. Antibiotic resistance is thus a major problem that threatens our ability to control even minor infections. It is therefore imperative that antibiotic stewardship is improved, and antibiotics are deployed in hospitals more effectively [17]. A further emerging issue with the use of antibiotics is an increased susceptibility to certain fungal infections. Antibiotics have been cited as an independent risk factor for the development of both mucosal and invasive *Candida* infections [18]. Mucosal candidiasis can occur as vulvovaginal candidiasis (VVC) or oropharyngeal candidiasis, and is associated specifically with the use of β-lactam antibiotics, whereas invasive candidiasis is mainly associated with the use of β-lactams, vancomycin and aminoglycosides [18,19]. VVC is thought to be linked to antibiotic use since these drugs deplete *Lactobacillus* species within the vagina. Lactobacilli are protective against *Candida* infection, as their secreted exopolysaccharides interfere with the growth, morphogenesis and adhesion of *C. albicans* to the vaginal epithelium—three vital components of disease [20]. Moreover, lactobacilli produce an aryl hydrocarbon receptor (AhR) ligand that induces AhR-dependent IL-22 transcription, a cytokine involved in barrier function and regulation of mucosal immune responses [21]. IL-22 is produced by innate lymphoid cells (ILCs) and is protective against VVC by regulating inappropriate neutrophil recruitment to the vaginal mucosa. This occurs via NLRC4-mediated production of the IL-1 receptor antagonist (IL-1Ra), which inhibits activation of the NLRP3 inflammasome and production of IL-1β, IL-1α and IL-18 (Figure 2) [22]. Thus, targeting excessive neutrophil responses in VVC can be therapeutic, since PMN depletion with an anti-Ly6G antibody or treatment with Anakinra (recombinant IL-1Ra) was found to be protective against VVC episodes, stressing the importance of IL-22 and neutrophils in the prevention of inflammation, dysbiosis and disease [22,23]. Indeed, human polymorphisms in *IL22* or *IDOL1* (the enzyme that catalyses IL-1Ra generation) are associated with VVC susceptibility, reinforcing the importance of this pathway in the protection against VVC [24].

For invasive candidiasis, antibiotics are thought to predispose individuals to this infection by significantly disrupting the microbiota, in particular by causing expansion of Proteobacteria and reductions in *Bifidobacterium* spp., Clostridial Firmicutes and Bacteroidetes [25], the latter of which are important for maintaining *C. albicans* colonisation resistance in mice by stimulating Hypoxia inducible factor 1α (HIF1α) activation and production of CRAMP, an antimicrobial peptide [26]. These antibiotic-induced perturbations in the microbiota create a niche availability for commensal *C. albicans* populations, and the resulting imbalance enhances the likelihood for invasive infection in immunocompromised individuals following disruption to the intestinal barrier caused by surgery, chemotherapy-induced mucositis and/or neutropenia [27]. Indeed, early studies showed that *Candida* isolated from the bloodstream of candidemic patients were the same as the isolates present in the patient’s GI tract, supporting the idea of an intestinal dysbiosis leading to a fungal bloom and subsequent dissemination via the bloodstream [28]. Due to the use of fluconazole prophylaxis in these at-risk patients, a shift in the causative agents of invasive candidiasis has occurred towards non-*albicans* species. For example, treatment with metronidazole, carbapenems, clindamycin and colistins have all been found to be strongly associated with disseminated *Candida glabrata* infections [19]. Unlike *C. albicans*, other non-*albicans* species have higher rates of fluconazole resistance [29]. Antibiotics may therefore favour colonisation by drug-resistant fungal isolates, or have mild antifungal effects, thus providing selection pressures for antifungal resistance to evolve [19]. 

In recent years, it has also been appreciated that antibiotics have additional consequences for the functioning of the mammalian immune system. For example, ciprofloxacin has been shown to suppress TLR expression, one of the key PRRs for anti-*Candida* defence [30], and its use in patients is significantly associated with invasive *Candida* infections [31]. Moreover, antibiotics alter the availability of adenosine monophosphate (AMP) within the intestine, which prevents AMPK activation of phagocytosis, and impairs the respiratory activity of macrophages, leading to an impairment of microbe killing [32,33]. A dysregulated metabolism can have significant consequences for antimicrobial immunity, since metabolic rewiring of immune cells is intimately linked with immune function. Upon LPS stimulation and fungal infection, macrophages switch to glycolysis for energy production, and the tricarboxylic acid (TCA) cycle subsequently stalls at two points, leading to the accumulation of the metabolites citrate and succinate. Citrate is used for membrane biogenesis, aiding lysosome formation and antigen presentation, while succinate accumulation leads to the sustained production of IL-1β and nitric oxide, two key components of antifungal immune defence [34]. Antibiotic-induced disturbance of immune-cell metabolism has now been described in several studies. For example, Kalghatgi and colleagues showed that quinolones, aminoglycosides and β-lactams disturbed the TCA cycle and electron transport chain within mitochondria, which led to the overproduction of ROS within immune cells, especially macrophages, leading to oxidative damage and hyper-responsiveness [35]. Indeed, intestinal macrophages within the gut are hyporesponsive and do not readily respond to PRR stimulation due to a high T_reg_ presence, thus preventing unwarranted inflammation and intestinal injury. However, following antibiotic treatment, intestinal macrophages shift into a state of hyper-responsiveness associated with increased inflammatory cytokine production and a T_h_1 bias, which impacted the ability of antibiotic-treated mice to control infections requiring T_h_2 and T_h_17-mediated responses [36]. These antibiotic-induced defects could be reversed by dietary addition of short-chain fatty acids (SCFAs), a microbial metabolite that becomes depleted during antibiotic treatment. SCFAs modulate a variety of immune functions, including PMN recruitment, DC maturation and T_reg_ differentiation [37,38]. In addition to depleting SCFA-producing bacteria, antibiotics have also been shown to impact other groups of bacteria important for mediating the T_h_17–T_reg_ balance in the intestine. Early work by Ivanov et al. [39] showed that segmented filamentous bacteria (SFB) are responsible for the modulation of inflammation within the gut, and are required for the development of T_h_17 cells. SFB elimination by antibiotics results in enhanced T_reg_ numbers, leading to an impaired T_h_17 response and enhanced susceptibility to *Citrobacter* infection [39,40]. T_h_17 responses are also important for antifungal defence at mucosal surfaces, since patients with *IL17RA* mutations are more susceptible to mucosal candidiasis [41]. T_h_17 cells promote neutrophil recruitment through the upregulation of *CXCL1* and *CXCL5*, and also stimulate antimicrobial peptide release to help combat fungal invasion [42]. 

Lastly, it has become increasingly appreciated that the effects of antibiotics on the immune system are not limited to the GI tract and extend to peripheral systems. For example, antibiotic-induced alteration of the maternal microbiota in mice has been shown to impair neutrophil progenitor development within neonates, which compromises their ability to fight bacterial sepsis [43]. Antibiotics have also been shown to affect immunity within the CNS, since antibiotics affected microglia maturation and function by the depletion of SCFAs within the gut [44]. This indicates that antibiotics can cause localised defects in mucosal immunity, as well as systemic effects; as such, there are profound consequences for protection against pathogenic microbes, including fungi.

## 4. Monoclonal Antibodies

Monoclonal antibodies are a class of biological drugs that target specific antigens, such as cytokines and their receptors, in order to block the action of these molecules and modulate inflammation. Monoclonal antibodies targeting IL-17A (such as ixekizumab and secukinumab) or IL-17RA (such as brodalumab) have been used in the treatment of autoimmune diseases, such as rheumatoid arthritis and psoriasis. However, as mentioned above, the IL-17 pathway is critical for antifungal host defence at mucosal barriers. In line with this, an increased frequency of mucosal candidiasis is observed in patients treated with these drugs [45,46]. Similarly, blocking antibodies specific for TNFα (e.g., infiliximab), which has proven to be an effective therapeutic strategy for patients with inflammatory bowel disease, are also associated with an increased susceptibility to fungal infections. In particular, GI candidiasis seems to be particularly prevalent [47], while infections caused by *Aspergillus* and *Histoplasma* species have also been observed [48].

## 5. Anti-Cancer Therapies

The face of cancer treatment has changed significantly over the last few decades, with a greater number of more targeted approaches becoming available. Despite these advances, individuals undergoing cancer treatment are still predisposed to a wide range of infections, including several caused by pathogenic fungi, which impacts the effectiveness of our modern-day cancer treatment regimens and the clinical outcome for these patients. Mechanistic insights into how these modern anti-cancer approaches affect antifungal immunity is still limited, and is an exciting new area of emerging research. Here, we outline some of the recent advances reported in this field. 

*Ibrutinib:* Ibrutinib is a recent addition to the arsenal of chronic lymphocytic leukaemia (CLL) treatment. Ibrutinib is an irreversible inhibitor of Bruton’s tyrosine kinase (Btk), which plays a key role in B-cell receptor signalling and induction of the NF-κB signalling pathway. Ibrutinib is an attractive treatment for cancer, as it has fewer infectious complications than other conventional chemotherapeutic agents, as it is not myelosuppressive and does not result in neutropenia [49,50]. However, several cases of fungal infections associated with the use of ibrutinib have now been reported in the literature. Specifically, ibrutinib appears to predispose individuals to invasive aspergillosis and *Pneumocystis* pneumonia within the first few months of treatment, while disseminated cryptococcosis has been reported as a late-onset infection [49,50]. These observations have led to the hypothesis that Btk may be a critical regulator of antifungal immunity. Recent studies support this, as ibrutinib treatment impairs the translocation of NFAT and NF-κB in human and murine macrophages stimulated with *Aspergillus* spores, thus disrupting Btk-mediated activation of NFAT signalling via a pathway that involved endosomal TLR9 [51]. Future work will need to determine the specific roles of Btk in antifungal immunity in response to different fungi and in different immune cell subsets, which may reveal new important pathways of protection and innate immune regulation that can be potentially utilised in the development of new antifungal treatments while preserving the benefits of ibrutinib.

*CXCR1/2 Inhibitors:* CXCR1 and CXCR2 are chemokine receptors that mediate neutrophil recruitment to the site of infection and activation of their antimicrobial activities. These receptors have also been linked to the survival and angiogenesis of melanoma cancers, hence they have become attractive targets for human melanoma therapy [52]. Indeed, CXCR1/2 inhibitors prevent metastasis of colonic cancer cells to the liver in a murine model, and have also been demonstrated to decrease the invasive potential of melanoma cells, indicating their effectiveness as therapeutics for metastatic cancers [52]. However, these chemokine receptors are also critical for the recruitment of neutrophils to fungal-infected organs. CXCR1 is highly induced in the *Candida*-infected kidney, and *Cxcr1*^−/−^ mice are unable to control *C. albicans* infection due to impaired neutrophil killing [53]. In humans, the mutant *CXCR1*-T276 allele is associated with an increased likelihood of *Candida* infection, caused by defective neutrophil degranulation and killing [53]. In patients and mice deficient in CARD9, defective production of CXCR1/2 ligands in the brain leads to decreased neutrophil trafficking and an unchallenged invasive fungal infection in the CNS [54], further underscoring the importance of CXCR1/2 signalling in the control of systemic *C. albicans* infections. These studies therefore indicate that CXCR1/2 inhibition could be a potential risk factor for the development of invasive candidiasis, and warrants the careful observation of patients on CXCR1/2 inhibitors for any signs of disseminated fungal infection [53], especially since CXCR1/2 are thought to play other non-conventional roles in immunity, including cell survival, proliferation and differentiation [53].

*Syk Inhibitors:* In addition to its role in CLR signalling and antifungal immunity, Syk kinase is also involved in tumour biology, both as a tumour activator (in B-cell malignancies) and suppressor (in acute myeloid leukaemia and Epstein–Barr virus-derived tumours) through its ability to modulate apoptosis, cell migration and tumorigenesis, depending on cell type [55]. Syk inhibitors have therefore been trialled as a potential new therapy for chronic lymphoid leukaemia and other haematological malignancies [56]. Syk inhibitors are likely to have a detrimental effect on antifungal immunity due to the central role of this kinase in fungal detection pathways (Figure 1). Syk deletion in murine DCs causes an enhanced susceptibility to *C. albicans* infection, through impaired IL-23 release. The absence of IL-23 causes the abrogation of GM-CSF secretion by natural killer (NK) cells, resulting in reduced neutrophil-mediated killing [57]. Syk activation is also essential for inflammasome activation and IL-1β secretion, which plays an important protective role against several pathogenic fungi, including *Aspergillus* and *Candida* species [58,59]. Deletion of the cytokine receptor IL-1R results in an increased fungal burden within a murine pulmonary histoplasmosis model, due to an increased T_h_2 response and downregulation of IFNγ production [60]. As with CXCR1/2 inhibitors, caution will need to be exercised when Syk inhibitor trials begin, in order to determine their potential effects on human antifungal immunity.

## 6. Conclusions

As outlined in this review, there are several controllable risk factors for fungal infections, and it will be important to determine the mechanisms underlying how these iatrogenic interventions cause an enhanced susceptibility to fungal infections. In the meantime, treatment with new immune-modulating drugs, including those discussed here, should be combined with a high degree of clinical suspicion regarding potential fungal infections to ensure early diagnosis and initiation of antifungal therapy. Fungal infections currently remain a significant clinical challenge, impacted by the difficulties in generating new antifungal compounds, a lack of fungal vaccines and the rising threat of antifungal drug resistance. Early diagnosis is key for the successful treatment of most fungal infections; hence, we urgently need more rapid and reliable diagnostic tests for fungal infections. Although there are several new drugs that have contributed towards the rise of human fungal infections in modern medicine, there are some anti-cancer drugs that have been found to additionally protect against fungal diseases. Checkpoint inhibitors (CPIs) are a promising treatment for haematological malignancies and target immunosuppressive molecules on T-cells such as PD-1 and CTLA-4, thus enhancing anti-tumour immunity [61], but may also enhance T-cell responses to pathogenic fungi. Interestingly, CPIs have been shown to protect against numerous fungal infections in murine models, including invasive candidiasis, disseminated cryptococcosis and histoplasmosis. Moreover, CPIs have been successfully used to reverse refractory mucormycosis in a patient, indicating that CPIs may have beneficial antifungal functions in humans [61,62]. Therefore, studying how CPIs and other new therapeutics affect antifungal immunity can lead to a greater understanding of mechanisms of protection, which will underpin novel therapeutics and diagnostics for fungal infections, which are sorely needed.
